# Supplementary material for: miR-150-5p and let-7b-5p in Blood Myeloid Extracellular Vesicles Track Cognitive Symptoms in Patients with Multiple Sclerosis
Source: Cells. 2022 May 5;11(9):1551. doi: 10.3390/cells11091551 (PMC9104242; doi:10.3390/cells11091551)
Supplement: Supplementary file 1 [file cells-11-01551-s001.zip › cells-1707084-supplementary.pdf]

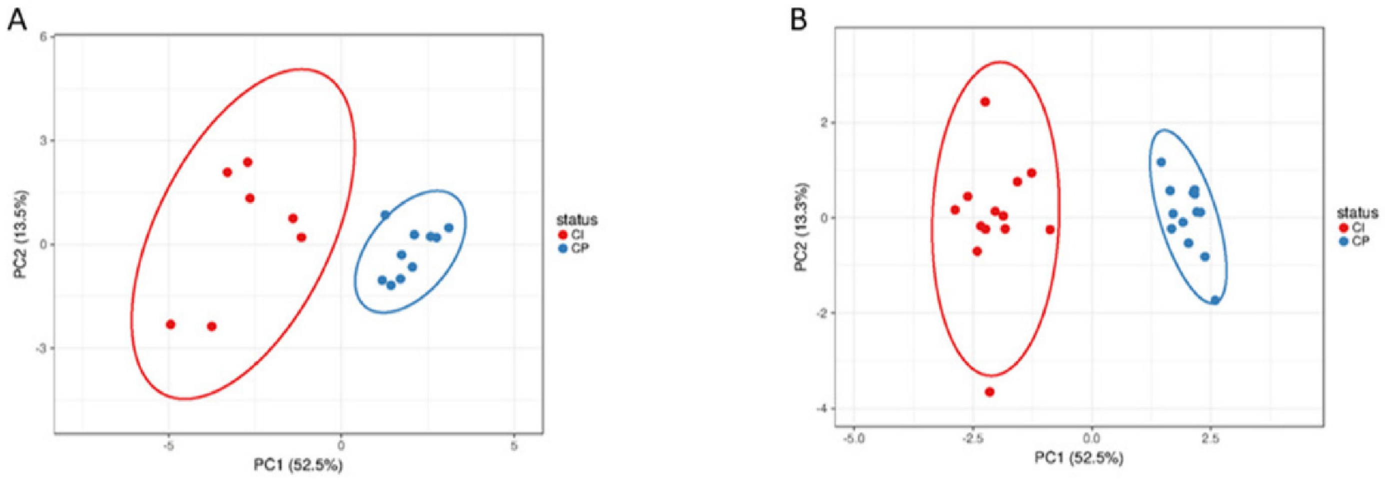

Figure S1 – Principal component analysis based on the selected miRNAs in IB4 positive EVs for **A)** Italian cohort and **B)** Amsterdam cohort.

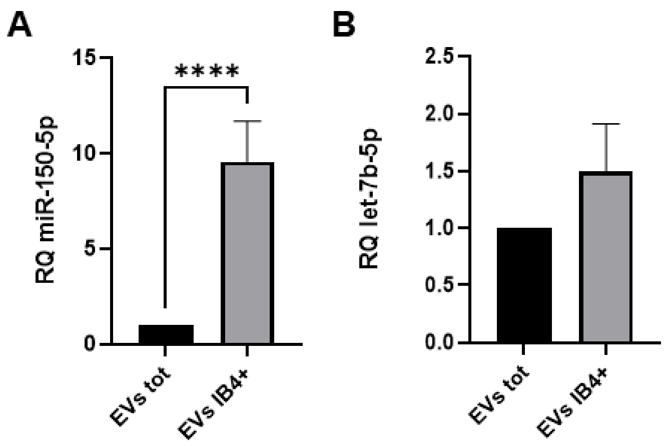

Figure S2 - miR-150-5p (**A**) and let-7b-5p (**B**) content in IB4 positive EVs compared to total EVs from the same patients.

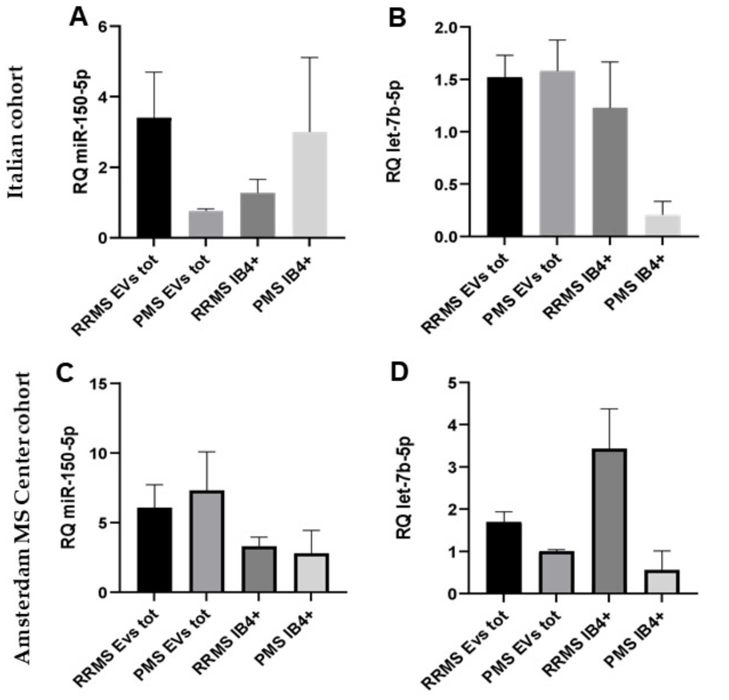

Figure S3 - Relative quantification of miR-150-5p **(A,C)** and let-7b-5p **(B, D)** in total EVs and IB4-positive EVs from RRMS versus PMS patients.

Table S1. – Gender distribution among CI and CP RRMS and PMS patients in the two cohorts.

	Disease type (gender)	Cognitive impaired (CI)	Cognitive Preserved (CP)
**Italian cohort**	RRMS (F:M)	6 (4:2)	11 (all Females)
	PMS (F:M)	4 (1:3)	0
**Amsterdam MS center cohort**	RRMS (F:M)	9 (8:1)	14 (12:2)
	PMS (F:M)	4 (2:2)	1 (Male)

Table S2. - miRNAs present in plasma EVs and their validated key synaptic targets.

miRNAs present in plasma EVs *	Validated key synaptic target
**let-7a-3p**	GABRG1, GSK3B, SEMA4D
**let-7b-5p**	AMPH, CACNG8, CPEB1, CPEB3, CPEB4, DBNL, DISC1, DNM3, EPHA4, FXR2, GNAS, GSK3A, HTT, KCNC4, KCTD12, NPTN, RAB40C, SEMA4C, SEPT4, SLC12A7, SNAP23, STRN, STX3, SYNGR2, SYNJ2BP, SYT1, TIAM1, UNC13B
**let-7g-5p**	DISC1, EPHA4, PLXND1, RAB40C, SEMA4C, SLC12A7, STRN, STX3, SYNJ2BP, SYT1
**miR-15a-5p**	BDNF, CLCN3, CPEB2, CPEB3, DLGAP3, EFNB2, GNAL, GSK3B, KIF1A, KPNA1, MINK1, PAK2, RAB40B, RIMS3, SNCG, SNTB2, STXBP3, SYPL1, WNT3A
**miR-15b-5p**	CPEB2, CPEB3, DLGAP3, GSK3B, KPNA1, MINK1, PAK2, RAB40B, RIMS3, SNTB2, STXBP1, STXBP3, SYNJ1, SYPL1
**miR-16-5p**	ADGRL1, AGRN, BDNF, CACNB2, CPEB2, CPEB3, DLGAP3 DAP3, EFNB2, GNAL, GSK3B, HTT SLC6A4, KCNC4, KIF5A, KPNA1, LIN7C, MINK1, NSF, PAK2, RAB12, RAB40B, RIMS3, SEMA4C, SNCG, SNTB2, STXBP3, SYNJ1, SYPL1, USP8, WNT3A, WNT5A
**miR-17-5p**	ADD1, EPHA4, FXR2, GNB5, GPM6A, HIP1, HTT SLC6A4, KCNMA1, KIF1A, PDZD11, PTPRO, RAB12, RAD21, SEMA4B, SLC12A6, SNAP47, SNTB2, STX6, SYNJ2BP, TPRG1L
**miR-19a-3p**	DBN1, FXR1, HOMER1, NAPB, NPTN, RAB13, RAB8B, RAP1A, RAP1B, SEMA4C, SLC12A7, STX12, STX6, TPRG1L, USP8

**miR-20a**	ADD1, ADGRL3, CDC42, CDK16, DLGAP2, EPHA4, FXR1, GNAS, GNB5, HIP1, LYPD6, MINK1, PDZD11, PTPRO, RAB12, SEMA4B, SLC12A6, SLC6A4, SLK, SNAP47, STX1B, STX6, SYNJ2BP, TPRG1L
**miR-21-5p**	CLCN5, CPEB3, DLG1, ENAH, EPHA4, FMR1, GNAQ, ITSN2, LIN7C, NTF3, PALLD, PPFIA4, PTK2, SEMA5A, SLKTIAM1, TPRG1L, WNT5A
**miR-23a-3p**	ADD3, MAP1B, NLGN4X, SLC6A6
**miR-24-3p**	ADD1, ADD3, AKAP5, CLSTN1, GABRB3, GPC4, SLC6A4, SNTB1
**miR-25-3p**	CLTA, CPEB1, CPEB3, CPEB4, GNAQ, KCN4, MAP1B, RAB3D, SEMA4C, SLC12A4, SLC12A5, SNTB2, STXBP1, SYNPR
**miR-26b-5p**	ADGRL2, AKAP5, ARHGAP44, CACNG3, CLSTN1, CLSTN2, CLTA, DBNL, DGKI, DGKZ, DLGAP4, DNAJC6, DTNB, GABRA3, GABRE, GABRG3, GPC4, GRIK2, GRM8, GSK3B, HTT, KCNQ2, KIF1B, L1CAM, LRFN3, MAP1B, MCTP2, NLGN4X, OR10H2, OR10H5, PNOC, PTPRO, RAB3A, RAB40B, RAB8A, SAMD14, SLC17A6, SNAP91, STX12, STX1A, SV2B, UNC13A
**miR-27a-3p**	ADD1, ADGRL1, CPEB4, GNB5, GSK3B, ITSN2, KCTD12, MAP1B, PAK2, PLXND1, RAP1B, SLC6A17, SNCG
**miR-30a-5p**	ABL1, ANXA1, BCL11A, BDNF, CDH2, CPEB4, DAG1, FBXO45, FXR1, FXR2, KIF5B, KPNA1, LIN7C, NDEL1, PICALM, RAP1B, SLC12A4, STRN, STX12, STX7, SYPL1, TPRG1L, WNT5A
**miR-92a-3p**	ADAM10, CDK16, CLTA, CPEB2, CPEB3, CPEB4, DNM2, EPHA4, EPHA7, EPHB2, FBXO45, FCHO2, FXR1, GNAQ, GSK3B, KCNC4, KIF1A, KIF5B, MAP1B, MINK1, NPTN, NSF, PLCG1, PSMA7, RAB3D, RAB8A, RAB8B, SLC12A4, SLC12A5, STX3, SV2A, SYNJ1, SYNPR
**miR-92b-3p**	ADAM10, CDK16, CLTA, CPEB2, CPEB3, CPEB4, EFNB1, FCHO2, FXR1, GNAQ, KCNC4, KIF1B, KIF5B, MAP1B, NPTN, NSF, PSMA7, RAB3D, RAB8B, RAD21, SLC12A5, SYNJ1
**miR-99a-3p**	No targets
**miR-99a-5p**	GLUD1, GNAL, SYNJ2BP
**miR-100-3p**	ACTBL2, GABRB1, PACSIN1, SYPL1, SYT9
**miR-101-3p**	CPEB1, FMR1, GRIK3, LIN7C, PAK3, RAB5A, RAB8B, RAC1, RAP1B, RHOA, WNT7A
**miR-122-5p**	ADAM10, AKAP5, ANK2, C14orf39, FAM9B, KCMF1, NLGN3, PPP1R9B, RAC1, RAD21, RHOA, SLITRK3, SNTB2, STXBP2, UNC13A
**miR-125a-5p**	CORO1C, HRH4, NDEL1, NTRK3, SEMA4B, SEMA4C
**miR-126-5p**	GABRA4, GLUD1, GRIK5, ITSN2, SLC12A6
**miR-142-3p**	CHRNA6, CLTA, CPEB2, DAG1, ENAH, GNAQ, GNAS, GPC4, KCTD12, KIF5A, KIF5B, PPFIA1, RAC1, STX2, STX6, SYPL1, SYT4
**miR-144-3p**	GRIK3, ITSN2, RAC1, WNT7A
**miR-146a-5p**	ACTBL2, MX2, NOS1, RAC1, RHOA, STON2, SYT12
**miR-146b-5p**	ACTBL2, RHOA
**miR-148a-3p**	CHRFAM7A, FXR1, GNB5, RAB12, SLC12A7, SNAPIN, STX6
**miR-149-5p**	ABL1, ARHGAP22, DBN1, FXR1, GPC1, GSK3A, ITSN2, KIF1A, OBSL1
**miR-150-5p**	EPBH2, GNB5, GRM6, HIP1, PAK3, PTCHD1, RAB13, RHOH, SCN2B, SLC6A4, SYNJ2BP
**miR-181a-3p**	No targets
**miR-181a-5p**	CADPS2, CHRFAM7A, CPEB4, ENAH, KPNA1, PPP1R9A, RAP1B, STX2, WNT3A
**miR-181c-5p**	CADPS2, CPEB4, KPNA1, RAP1B, SCN8A, STX2
**miR-185-3p**	ABL1, SEMA3F, SYNGR2
**miR-185-5p**	ADD2, CDC42, CTTN, DLGAP4, EPHB2, GLUD1, LY6H, NTRK3, RAB40C, RHOA, SHISA9, SLC6A17, SLC8A1, STX6, SYT2, SYT7
**miR-186-5p**	CACNB1, CAMK2B, DAGLB, DBN1, DLG3, GRIK5, GUCY1B3, ITSN2, KCTD12, KIF5B, KPNA1, MINK1, RAB12, RIMS3, RIMS4, SCFD1, SLC6A6, SYNJ2BP, TUBB2B, WNT5A
**miR-192-5p**	CADPS2, CLSTN1, EFNB2, EMB, GPC4, GRIA1, HRH1, KCNC3, KCTD12, KIF5B, KIF5C, LHFPL4, NSF, PDE2A, PPAT, RAB3B, RAB40B, RAB8B, STX7, SYNGR1
**miR-199a-3p**	SYNGR2, GNAS

**miR-204-5p**	CACNG8, CAMK2G, CDC42, CHD2, CDH8, EFNB2, ENAH, EPHA4, HTR1F, LY6G6D, MCTP1, MX1, NTRK2, PLCG1, RAB40B, SYNGR1, SYNJ2BP, SYT6, UNC13D
**miR-221-3p**	ADD1, ANXA1, FMR1, RHOA, STN1, TIAM1, UNC13B
**miR-223-3p**	CACNG8, SLC12A7, SYNGR2
**miR-320a**	ACTBL2, ADD1, ARHGEF2, CPEB3, EPHA4, GNAS, NET1, RAC1, SEPT1, SYNGR2, SYNJ2BP, UNC13D
**miR-340-5p**	BCL11A, CTTN, FCHO2, NET1, RHOA, RIMS4, SLC24A2, SLITRK4, SYPL1
**miR-345-5p**	NTRK3
**miR-376a-3p**	GNAS, KIF5C, RIMS2, STON2
**miR-451a**	ADAM10, RAB5A
**miR-452-3p**	RAB3B
**miR-483-5p**	CACNG8, GPC6, RHOA, STX6, SYT7
**miR-484**	ABL1, ACP4, ADAM10, BSN, CDC42, EFNB3, GRIA4, GRIPAP1, HCLS1, HOMER1, LIN7C, RAB40C, RAC3, SEMA4D, SLC6A8, SLK, STRN, SYNGR2, SYNJ2BP
**miR-487a-3p**	CPEB4, MAGI2, MAP1B, RAB3B, SYPL1
**miR-490-3p**	DISC1, RHOA, SYT15
**miR-501-3p**	NLGN4X
**miR-516b-5p**	CLCN3, GNAS, PLCG1
**miR-576-3p**	EMB, STON2
**miR-652-3p**	CACNG8, KIF1A, RAC1, SLC12A6
**miR-1283**	GLUD2

* Ref. [31].

Table S3. - Selected miRNAs and their validated key synaptic targets.

**Selected miRNAs**	**Validated key synaptic target**
**miR-146a-5p**	ACTBL2, MX2, NOS1, RAC1, RHOA, STON2, SYT12
**miR-223-3p**	CACNG8, SLC12A7, SYNGR2
**miR-16-5p**	ADGRL1, AGRN, BDNF, CACNB2, CPEB2, CPEB3, DLGAP3 DAP3, EFNB2, GNAL, GSK3B, HTT SLC6A4, KCNC4, KIF5A, KPNA1, LIN7C, MINK1, NSF, PAK2, RAB12, RAB40B, RIMS3, SEMA4C, SNCG, SNTB2, STXBP3, SYNJ1, SYPL1, USP8, WNT3A, WNT5A
**miR-23a-3p**	ADD3, MAP1B, NLGN4X, SLC6A6
**miR-20a**	ADD1, ADGRL3, CDC42, CDK16, DLGAP2, EPHA4, FXR1, GNAS, GNB5, HIP1, LYPD6,<br>MINK1, PDZD11, PTPRO, RAB12, SEMA4B, SLC12A6, SLC6A4, SLK, SNAP47, STX1B, STX6, SYNJ2BP, TPRG1L
**miR-21-5p**	CLCN5, CPEB3, DLG1, ENAH, EPHA4, FMR1, GNAQ, ITSN2, LIN7C, NTF3, PALLD, PPFIA4, PTK2, SEMA5A, SLKTIAM1, TPRG1L, WNT5A
**miR-125a-5p**	CORO1C, HRH4, NDEL1, NTRK3, SEMA4B, SEMA4C
**miR-146b-5p**	ACTBL2, RHOA
**miR-150-5p**	EPBH2, GNB5, GRM6, HIP1, PAK3, PTCHD1, RAB13, RHOH, SCN2B, SLC6A4, SYNJ2BP
**miR-24-3p**	ADD1, ADD3, AKAP5, CLSTN1, GABRB3, GPC4, SLC6A4, SNTB1
**miR-15a-5p**	BDNF, CLCN3, CPEB2, CPEB3, DLGAP3, EFNB2, GNAL, GSK3B, KIF1A, KPNA1, MINK1, PAK2, RAB40B, RIMS3, SNCG, SNTB2, STXBP3, SYPL1, WNT3A
**let-7b-5p**	AMPH, CACNG8, CPEB1, CPEB3, CPEB4, DBNL, DISC1, DNM3, EPHA4, FXR2, GNAS, GSK3A, HTT, KCNC4, KCTD12, NPTN, RAB40C, SEMA4C, SEPT4, SLC12A7, SNAP23, STRN, STX3, SYNGR2, SYNJ2BP, SYT1, TIAM1, UNC13B
**miR-126-5p**	GABRA4, GLUD1, GRIK5, ITSN2, SLC12A6
**miR-451a**	ADAM10, RAB5A

Table S4. - Correlation analysis among clinical variables and miR150-5p and let7b-5p levels.

Correlation Spearman r	miR-150-5p EVs tot	Let-7b-5p EVs tot	miR-150-5p IB4+ EVs	Let-7b-5p IB4+ EVs	Age	DoD	AAO	EDSS	sNf-L
**miR-150-5p EVs tot**	1								
**Let-7b-5p EVs tot**	0.43	1							
**miR-150-5p IB4+ EVs**	-0.19	-0.12	1						
**Let-7b-5p IB4+ EVs**	-0.22	-0.24	0.32	1					
**Age**	-0.29	0.09	-0.05	-0.04	1				
**DoD**	-0.25	-0.19	-0.07	-0.07	0.49	1			
**AAO**	-0.33	0.12	0.06	-0.08	0.93	0.36	1		
**EDSS**	-0.21	-0.10	-0.03	-0.08	0.34	0.78**	0.24	1	
**sNf-L**	-0.13	-0.03	0.46*	0.45	0.43	0.47	0.33	0.15	1

Spearman correlation analysis between candidate miRNAs levels and clinical variables in total MS Italian population **P<0.001, *P=0.04

Correlation Spearman r	miR-150-5p EVs tot	Let-7b-5p EVs tot	miR-150-5p IB4+ EVs	Let-7b-5p IB4+ EVs	Age	DoD	AAO	EDSS	sNf-L
**miR-150-5p EVs tot**	1								
**Let-7b-5p EVs tot**	0.72	1							
**miR-150-5p IB4+ EVs**	-0.20	-0.06	1						
**Let-7b-5p IB4+ EVs**	0.21	-0.32	0.36	1					
**Age**	0.15	0.09	-0.21	0	1				
**DoD**	-0.22	0.31	-0.14	-0.95	-0.01	1			
**AAO**	-0.12	-0.13	-0.03	-0.20	0.88	0.08	1		
**EDSS**	-0.29	-0.11	0.17	-0.66	0.30	0.81**	0.27	1	
**sNf-L**	-0.28	0	-0.54	0.10	0.36	-0.09	0.07	0.14	1

Spearman correlation analysis between candidate miRNAs levels and clinical variables in CI MS Italian cohort, **P<0.001

Correlation Spearman r	miR-150-5p EVs tot	Let-7b-5p EVs tot	miR-150-5p IB4+ EVs	Let-7b-5p IB4+ EVs	Age	DoD	AAO	EDSS	sNf-L
**miR-150-5p EVs tot**	1								
**Let-7b-5p EVs tot**	0.21	1							
**miR-150-5p IB4+ EVs**	-0.14	-0.03	1						
**Let-7b-5p IB4+ EVs**	-0.18	-0.31	0.32	1					
**Age**	-0.26	0.33	-0.11	-0.51	1				
**DoD**	0.09	-0.37	-0.48	-0.40	-0.05	1			
**AAO**	-0.36	0.41	-0.04	-0.40	0.97	-0.15	1		
**EDSS**	0.23	0.08	-0.14	-0.27	-0.40	0.16	-0.38	1	
**sNf-L**	-0.22	-0.20	0.61	0.50	0.30	0.11	0.36	-0.40	1

Spearman correlation analysis between candidate miRNAs levels and clinical variables in CP MS Italian cohort.

Correlation Spearman r	miR-150-5p EVs tot	Let-7b-5p EVs tot	miR-150-5p IB4+ EVs	Let-7b-5p IB4+ EVs	Age	DoD	AAO	EDSS	sNf-L
**miR-150-5p EVs tot**	1								

**Let-7b-5p EVs tot**	-0.39	1							
**miR-150-5p IB4+ EVs**	0.22	0.12	1						
**Let-7b-5p IB4+ EVs**	0.01	0.50	0.27	1					
**Age**	-0.01	0.02	0.10	-0.31	1				
**DoD**	0.23	-0.03	-0.05	0.30	0.55	1			
**AAO**	-0.08	0.02	0.07	0.04	0.54	0.13	1		
**EDSS**	0.03	-0.33	-0.07	-0.17	0.18	0.04	0.04	1	
**sNf-L**	-0.01	-0.02	0.47*	-0.28	0.48	0.19	0.24	0.31	1

Spearman correlation analysis between candidate miRNAs levels and clinical variables in total MS Amsterdam Center population, *P=0.04

**Correlation Spearman r**	**miR-150-5p EVs tot**	**Let-7b-5p EVs tot**	**miR-150-5p IB4+ EVs**	**Let-7b-5p IB4+ EVs**	**Age**	**DoD**	**AAO**	**EDSS**	**sNf-L**
**miR-150-5p EVs tot**	1								
**Let-7b-5p EVs tot**	-0.37	1							
**miR-150-5p IB4+ EVs**	0.78	-0.2	1						
**Let-7b-5p IB4+ EVs**	0.23	-0.27	0.52	1					
**Age**	0.42	0.27	0.41	-0.08	1				
**DoD**	0.42	-0.22	0.16	-0.19	0.67**	1			
**AAO**	-0.67	-0.36	-0.55	-0.10	-0.26	-0.19	1		
**EDSS**	0.33	0.63	0.27	0.29	0.21	0.16	-0.46	1	
**sNf-L**	0.30	-0.10	0.32	-0.09	0.34	-0.18	-0.09	0.19	1

Spearman correlation analysis between candidate miRNAs levels and clinical variables in CI MS Amsterdam Center cohort, **P<0.01

**Correlation Spearman r**	**miR-150-5p EVs tot**	**Let-7b-5p EVs tot**	**miR-150-5p IB4+ EVs**	**Let-7b-5p IB4+ EVs**	**Age**	**DoD**	**AAO**	**EDSS**	**sNf-L**
**miR-150-5p EVs tot**	-0.39	1							
**Let-7b-5p EVs tot**	-0.29	0.50	1						
**miR-150-5p IB4+ EVs**	-0.17	0.60	0.31	1					
**Let-7b-5p IB4+ EVs**	-0.25	0.42	-0.09	-0.19	1				
**Age**	0.21	0.22	-0.27	-0.30	0.35	1			
**DoD**	-0.14	0.72	-0.02	-0.02	0.31	0.24	1		
**AAO**	-0.13	-0.14	-0.03	-0.06	0.04	-0.08	0.14	1	
**EDSS**	-0.50	0.34	0.47	0.22	0.40	-0.02	0.37	0.22	1
**sNf-L**									

Spearman correlation analysis between candidate miRNAs levels and clinical variables in CP MS Amsterdam Center cohort.

Abbreviations: EVs tot = total Extracellular Vesicles; IB4+ EVs = IB4 positive Extracellular Vesicles; DoD = duration of disease; AAO = age at disease onset; MS = Multiple sclerosis; EDSS = Expanded Disability Status Scale; sNf-L = neurofilament serum levels.
